# Marker‐assisted forward breeding to develop a drought‐, bacterial‐leaf‐blight‐, and blast‐resistant rice cultivar

**DOI:** 10.1002/tpg2.20170

**Published:** 2021-11-29

**Authors:** Uma Maheshwar Singh, Shilpi Dixit, Shamshad Alam, Shailesh Yadav, Vinukonda Vishnu Prasanth, Arun Kumar Singh, Challa Venkateshwarlu, Ragavendran Abbai, Abhilash Kumar Vipparla, Jyothi Badri, Tilatoo Ram, Madamshetty Srinivas Prasad, Gouri Sankar Laha, Vikas Kumar Singh, Arvind Kumar

**Affiliations:** ^1^ International Rice Research Institute South Asia Hub, ICRISAT Patancheru Hyderabad India; ^2^ International Rice Research Institute South Asia Regional Centre Varanasi India; ^3^ ICAR‐Indian Institute of Rice Research Rajendra Nagar Hyderabad India; ^4^ International Crops Research Institute for the Semi‐Arid Tropics Patancheru Hyderabad India

## Abstract

Among the different challenges related to rice (*Oryza sativa* L.) cultivation, drought, bacterial leaf blight (BLB), and blast are the key stresses that significantly affect grain yield (GY) in rice. To ameliorate this issue, marker‐assisted forward breeding (MAFB) coupled with a simultaneous crossing approach was used to combine three drought tolerant quantitative trait loci (QTL)—*qDTY_1.1_*, *qDTY_3.1_*, and *qDTY_12.1_*—four BLB genes—*Xa4*, *xa5*, *xa13*, and *Xa21*—and one blast‐resistance gene, *Pi9*, in the elite rice cultivar Lalat. The introgression lines (ILs) developed in the current study were phenotypically screened for drought, BLB, and blast resistance at the F_7_–F_8_ generation. Under the reproductive stage (RS) drought stress, the yield advantage of ILs, with major‐effect QTL (*qDTY*) over elite parent Lalat, ranges from 9 to 124% in DS2019 and from 7 to 175% in WS2019. The selected ILs were highly resistant to BLB, with lesion lengths ranging from 1.3 to 3.0 cm and blast scores ranging from 1 to 3. ILs that were tolerant to RS drought, resistant to BLB, and blast disease and had similar or higher yields than Lalat were analyzed for grain quality. Six ILs were found to have similar grain quality characteristics to Lalat including hulling, milling, head rice recovery (HRR), chalkiness, alkali spreading value (ASV), and amylose content (AC). This study showed that MAFB, together with simultaneous crossing, would be an effective strategy to rapidly combine multiple stresses in rice. The ILs developed in this study could help to ensure yield sustainability in rainfed environments or be used as genetic material in future breeding programs.

## Core Ideas

- Changing climatic conditions have increased the occurrence of various stresses in rice.
- Marker‐assisted forward breeding is a quick and effective method for combining multiple QTL and genes.
- Lines with 5–8 QTL and genes for drought and genes for blast and bacterial leaf blight were developed.
- Lines having multiple QTL and genes reduce yield loss caused by different stresses.

**Abbreviations**
AC: amylose content
ASV: alkali spreading value
BLB: bacterial leaf blight
DFF: days to 50% flowering
DS: dry season
GY: grain yield
HRR: head rice recovery
IL: introgression lines
IRRI: International Rice Research Institute
KB: kernel breadth
KL: kernel length
L/B: kernel length/breadth ratio
MABB: marker‐assisted backcross breeding
MAFB: marker‐assisted forward breeding
NS: nonstress
PCR: polymerase chain reaction
*qDTY*
: major‐effect QTL
QTL: quantitative trait loci
RS: reproductive stage
SES: standard evaluation system
SNP: single‐nucleotide polymorphism
SS: severe drought stress
WS: wet season.

## 1 INTRODUCTION

Climate change has drastically affected agriculture and is a major challenge to sustainable rice (*Oryza sativa* L.) production. Continuous changes in climatic patterns have the potential to threaten the vulnerable world's food security in many ways including exacerbating major diseases and pests and creating favorable climatic conditions for the emergence of devastating new diseases and race, increased drought, and other abiotic stresses at various growth stage in critical food‐producing regions (Velásquez et al., 2018). Rice, being an important and most widely consumed cereal crop, is severely affected by biotic and abiotic stresses (Singh et al., 2016). The occurrences of multiple abiotic and biotic stresses have demanded development of climate‐smart rice by combining quantitative trait loci (QTL) and genes for tolerance or resistance in the high‐yielding cultivars to confer a wider range of tolerance or resistance. Enhanced capability of climate‐smart cultivars would enable the crop to thrive under adverse environmental conditions. The recent advances in molecular marker technology and genomics have played an important role in developing single‐ and multiple‐stress‐tolerant rice cultivars (Singh et al., 2014). The availability of linked and gene‐specific markers for QTL and genes for different abiotic and biotic stresses has provided the opportunity to rapidly stack multiple QTL and genes quickly in popular high‐yielding cultivars.

The introduction of semi‐dwarf rice cultivars, widespread usage of high responsive inputs such as fertilizers, and insecticides has increased production (Conway G, 2020); and India has become the world's second‐largest rice producer (http://www.fao.org/faostat/en/#home). However, these high‐yielding cultivars are susceptible to most of the abiotic and biotic stresses, which makes rice production vulnerable mainly during years of high disease infestation or abiotic stress occurrence, which are very frequent in current scenarios because of changing climatic conditions. Abiotic stress, such as drought, and biotic stress, such as bacterial leaf blight (BLB) and blast, which are a serious threat in many rice growing places, particularly in India, are the most common stresses that cause major yield losses.

Drought is one of the most serious climatic challenges to the rainfed area of rice cultivation in southern and southeastern Asia, affecting >23 million ha of rice area (Pandey et al., 2006). Although drought affects rice growth adversely in all the stages of plant growth, reproductive stage (RS) being most sensitive to drought stress, drought has resulted in significant yield loss (Barnabas et al., 2008). Through 2014, at the International Rice Research Institute (IRRI), 14 major‐effect QTL (*qDTY*) have been identified for grain yield (GY) under drought, for example, *qDTY_1.1_*, *qDTY_2.1_*, *qDTY_3.1_*, *qDTY_6.1_*, *qDTY_3.2_*, *qDTY_12.1_*, *qDTY_2.2_*, *qDTY_4.1_*, *qDTY_9.1_* and *qDTY_10.1_* (Mishra et al., 2013; Swamy et al., 2013; Venuprasad et al., 2009; Venuprasad et al., 2012; Yadaw et al., 2013). Several *qDTY* have been effectively used in modern drought breeding programs. These *qDTY* have resulted in yield advantage of 150–500 kg ha^−1^, with genetic gain of 10–30% under RS drought conditions. Recently, Sandhu et al., 2019 introgressed QTL for drought and gene for submergence tolerance (*qDTY_1.1_* + *qDTY_2.1_* + *qDTY_3.1_* + *Sub1*) and found that the performance of near‐isogenic lines had yield advantage of 292–1,118 kg ha^−1^ and 284–2,086 kg ha^−1^ under RS drought stress, whereas 76–2,479 kg ha^−1^ and 396–2,376 kg ha^−1^ yield advantage was observed under nonstress (NS) over two consecutive seasons. Shamsudin et al., 2016 pyramided three *qDTY* (*qDTY_2.2_*, *qDTY_3.1_*, and *qDTY_12.1_*) in ‘MRQ74’ and ILs with yield advantages of 1,009–3473 kg ha^−1^ under RS, with yield equivalent to recurrent parent under NS trials was found. Dixit et al., 2017b introgressed two drought‐tolerant QTL (*qDTY_3.2_* and *qDTY_12.1_*) in ‘Sabitri’, and yield of >6,500 kg ha^−1^ under NS and 500–600 kg ha^−1^ under RS stress was achieved. Dixit et al., 2017a introgressed three QTL (*qDTY_3.1_*, *qDTY_6.1_*, and *qDTY_6.2_*) for drought and gene *Sub1* for submergence in ‘TDK1’ background, where yield of 37–300 kg ha^−1^ and 4,897–5,244 kg ha^−1^ was recorded under RS and NS, respectively.

**Core Ideas**

- Changing climatic conditions have increased the occurrence of various stresses in rice.
- Marker‐assisted forward breeding is a quick and effective method for combining multiple QTL and genes.
- Lines with 5–8 QTL and genes for drought and genes for blast and bacterial leaf blight were developed.
- Lines having multiple QTL and genes reduce yield loss caused by different stresses.

Among the biotic stress, BLB is one of the most severe and widespread diseases in rice caused by *Xanthomonas oryzae* pv. *oryzae*. It significantly reduces the rice yield with partial filling of grains because of restrictions on the limited photosynthetic area (Pradhan et al., 2015). The effective way to control BLB in rice by chemical use is not popular, therefore use of BLB‐resistant rice cultivars is the most effective and environmentally safe way to protect the crop from BLB disease (Khush et al., 1989). To date, more than 45 BLB resistance genes (Kim, 2018; Neelam et al, 2020) have been identified and 11 of them have been cloned and characterized (Wang et al, 2020). Some of them have been introduced into recent high‐yielding rice cultivars (Dokku et al., 2013a, 2013b; Joseph et al., 2004; Pradhan et al., 2015; Suh et al., 2013; Sundaram et al., 2008). The genes may differ in the level of resistance to a number of virulent pathogens. Thus, to combat the effect of BLB pathogens, gene pyramiding is currently being pursued to develop more durable resistant rice cultivars (Dokku et al., 2013a, 2013b; Huang et al., 1997; Pradhan et al., 2015; Suh et al., 2013).

Blast, caused by the fungus *Magnaporthe oryzae* Barr, is the most serious fungal disease in rice. Rice yield loss because of blast can be as high as 50% when the disease becomes epidemic (Babujee & Gnanamanickham, 2000). This loss can go up to 70–80% in severe cases. Up to now, more than 101 blast‐resistant genes and 350 QTL have been identified (Ashkani et al., 2015). Out of which, 31 genes [*Pi37*, *Pit*, *Pish*, *Pi35*, *Pi64*, *Pib*, *pi21*, *Pi63/Pikahei‐1(t)*, *Pid2*, *Pi9*, *Pi2*, *Pizt*, *Pid3*, *Pi25*, *Pi50*, *Pigm*, *Pid3‐I1*, *Pi36*, *Pi5*, *Pikm*, *Pb1*, *Pi54*, *Pia*, *Pikp*, *Pik*, *Pi1*, *Pi‐Co39*, *Pike*, *Pita*, *Ptr*] are cloned and molecularly characterized (Alam et al., 2017; Rajashekara et al., 2014; Sharma et al., 2012; Xiao Ning et al, 2020). Among these, a minimum of 14 genes [*Pi1*, *Pi2*, *Pi9*, *Pi20(t)*, *Pi33*, *Pi39*, *Pi40(t)*, *Pi47*, *Pi48*, *Pi54rh*, *Pi56*, *Piz*, *Piz*‐t, and *Pigm*] have been defined based on their wide‐scale resistance (Das et al., 2012; Hua et al., 2015, 2012; Huang et al., 2011; Liu et al., 2013). Many of the resistant genes have been incorporated in modern rice cultivars (Ashkani et al., 2015). Koide et al. (2010) has pyramided two blast resistance genes, *Pish* and *Pib*, after which higher resistance was achieved. Xiao et al. (2016) have pyramided two blast resistance genes (*Pi46* and *Pita*), which showed complementary effects between the genes. Two blast resistance genes (*Piz5* and *Pi54*) were pyramided into an elite Basmati restorer line PRR78, where a strong level of resistance was observed (Singh et al., 2013). Broad‐spectrum blast resistant gene *Pi‐9(t)* has been introgressed into the hybrid restorer Luhui17 by using marker‐assisted backcross breeding (MABB), where a higher level of resistance was observed (Wen & Gao, 2012). These studies indicate that the use of single, broad‐spectrum blast resistance genes or multiple gene pyramiding could be effective against blast disease.

Recently, efforts have been made to pyramid multiple QTL and genes for biotic and abiotic stress in rice (Das & Rao, 2015; Das et al., 2018; Dixit et al., 2020). Different breeding strategies were adopted for development of pyramided lines depending on the donor type, availability of QTL and genes, marker, genotyping facilities, time, etc. Previously, efforts have been made to combine multiple stress tolerance traits in rice, for instance, drought and submergence tolerance (Bhandari et al., 2019; Dixit et al., 2017a; Sandhu et al., 2019); BLB and blast resistance (Arunakumari et al., 2016; Balachiranjeevi et al., 2015; Fu et al., 2012; Hari et al., 2013; Narayanan et al., 2002; Pinta et al., 2013; Singh et al., 2012; Vipparla et al., 2016a, 2016b); brown planthopper, BLB, blast, rice stripe virus resistance (Reinke et al., 2018); BLB, blast, gall midge, submergence resistance or tolerance (Das et al., 2018); and BLB, blast, gall midge, salinity, submergence resistance or tolerance (Das & Rao, 2015). Based on the success of previous QTL–gene stacking studies, the present breeding effort was started to combine drought, BLB, and blast in elite parent ‘Lalat’. Simultaneous crossing has been used to combine traits from different donors, and MABF was deployed to track alleles at different generations.

## 2 MATERIALS AND METHODS

### 2.1 Plant materials and crossing scheme

This study was conducted at IRRI South Asia hub, located at ICRISAT, Patancheru, Hyderabad, India, (17.53° N, 78.27° E; 545 m asl). In this study, Lalat, a semi‐dwarf (85–90 cm) rice cultivar with a medium duration (120–125 d), was chosen as an elite parent. Lalat was released in 1987 for irrigated ecology of Odisha, West Bengal, and Jharkhand. It has long slender grains with average productivity of 4.0–4.5 t ha^−1^. In this breeding program, IR96321‐1447‐561‐B‐1 (*qDTY_1.1_*, *qDTY_3.1_*) and IR74371‐46‐1‐1 (*qDTY_12.1_*) were used as sources of QTL for RS drought tolerance. IRBB60, possessing a set of four genes (*Xa4*, *xa5*, *xa13*, *Xa21*) was used as a source of BLB resistance. Lalat itself possesses the *Xa4* gene (Dokku et al., 2013b). Similarly, IRBL9, possessing the *Pi9* gene in the japonica background, was used as a source of the blast resistance gene (Supplemental Table S1).

**FIGURE 1:**
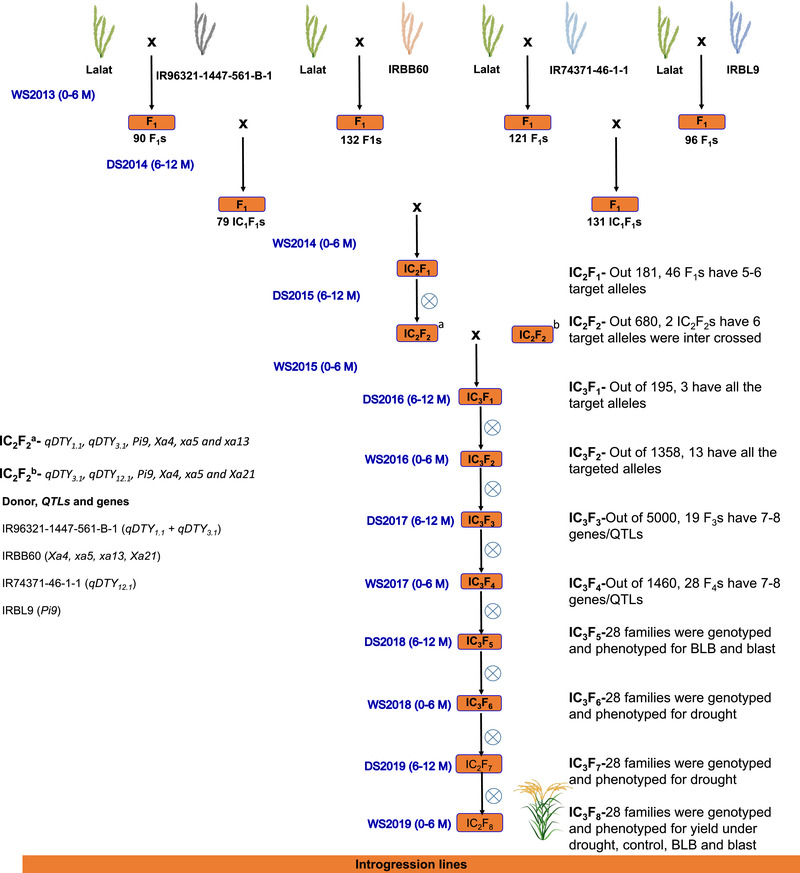
Crossing was started by adopting simultaneous and synchronized marker‐assisted breeding approach. In this, the recurrent parent ‘Lalat’ is first crossed with each of four donor parents to produce F_1_ progeny. Four of the single‐crossed F_1_ progeny are intercrossed with each other to produce two double‐cross IC_1_F_1_ populations and these two double‐cross IC_1_F_1_ populations are intercross again to produce IC_2_F_1_ progeny, which integrate all targeted genes and QTL in heterozygous state. The progeny is advanced to IC_2_F_2_ populations and to stack all the targeted QTL and genes in root genotype selected IC_2_F_2_ population are intercross and further advanced to IC_3_F_n_ populations through marker‐assisted forward breeding

To combine all the target QTL and genes in one genotype, simultaneous crossing with different donors was initiated during the wet season (WS) of 2013. A graphical representation of the marker‐assisted forward breeding (MAFB) scheme is adopted to combine all the targeted QTL and genes in elite parent Lalat (Figure 1). In this, Lalat was first crossed with four different donor parents to produce single cross F_1_ progeny. Four single‐cross true F_1_ progeny were intercrossed and double crossed with each other to produce two different intercrossed IC_1_F_1_ populations, and these two intercross IC_1_F_1_ populations were crossed again to produce a second intercross IC_2_F_1_, which integrates all eight targeted QTL and genes in the heterozygous state. The developed IC_2_F_1_ was advanced to a further generation based on MAFB (Figure 1). To achieve all eight targeted QTL and genes in a single genotype; two sets of IC_2_F_2_ populations were intercrossed and the generated IC_3_F_1_ progeny were advanced. The developed IC_3_F_1_ was advanced to further generations following the strategy of MAFB, which involved initial genotyping of the individual plant followed by selecting plants with maximum gene combinations together with good agronomic performance at every step of advancing the material (Figure 1).

### 2.2 Marker‐assisted foreground selection of ILs

Genomic DNA was extracted from young leaves of parents and ILs using the TPS method (Kim et al., 2016). For all three *qDTY* (*qDTY_1.1_*, *qDTY_3.1_*, and *qDTY_12.1_*) and five genes (*Xa4*, *xa5*, *xa13*, *Xa21*, and *Pi9*), polymerase chain reaction (PCR)‐based genotyping was performed. Here, *xa5*, *xa13*, *Xa21*, and *Pi9* are functional markers, whereas *Xa4* is a gene‐linked marker. Both peak and flanking markers were used for QTL, while gene‐specific and linked markers were used for genes. Parental polymorphism survey of QTL and genes was conducted, and markers showing polymorphism were used for genotyping (Supplemental Tables S1 and S2). The PCR was conducted in a total volume of 20 μl containing 50 ng of DNA template, 1 μl 10× PCR buffer, 0.25 μM of each primer, 75 μM of each dNTP, and 0.25 units of Taq DNA polymerase (Genei, India). The condition of PCR amplification included one cycle at 94 °C for 5 min, followed by 30 cycles of 94 °C for 30 s, 50∼60 °C for 30–60 s, 72 °C for 45–70 s (depending on the primers), and a final incubation at 72 °C for 5 min. The PCR products were analyzed on 2–4% agarose gel by electrophoresis.

Finally, selected ILs were genotyped with trait‐based single‐nucleotide polymorphism (SNP) markers developed by IRRI using Kompetitive allele‐specific PCR SNP assay with a genotypic service provider (Intertek, Hyderabad, India). The SNP markers linked to the traits [snpOS00400, snpOS00402, and snpOS00408 (*qDTY_1.1_*); snpOS00085, snpOS00086 and snpOS00089 (*qDTY_3_*_._ *_1_*); snpOS00483 and snpOS00484 (*qDTY_12_*_._ *_1_*); snpOS00481 (*Xa4*); snpOS00493 and snpOS494 (*xa13*); snpOS0061 (*Xa21*); and snpOS00451 (*Pi9*)] were used for genotyping (https://isl.irri.org/). Because the SNP marker for the *xa5* gene was not available, it was only confirmed with gene linked markers on agarose gel.

### 2.3 Breeding line selection strategies

All experiments were carried out at research fields of IRRI, South Asia Hub. The ILs were selected at various stages of MAFB, screened for blast and BLB at nursery and tillering stages, respectively. The resistant ILs were evaluated for drought tolerance under both RS and NS experimental conditions. The experiments were performed under lowland transplanted conditions in which 21‐d‐old seedlings were transplanted with one single seedling per hill in the well‐prepared field.

#### 2.3.1 Evaluation of ILs under NS and RS drought‐stress condition

The NS experiment was conducted during dry season (DS) 2019 and WS2019, where the selected ILs were planted in an augmented randomized complete block design with repeated checks (‘IR64’, ‘Sahbhagi dhan’), Lalat, and drought donor (IR74371‐46‐1‐1; IR96321‐1447‐561‐B‐1) in a plot size of 2.4 m^2^ for NS experiment. A single seedling per hill was planted in the field. The row‐to‐row and hill‐to‐hill spacing of 20 and 15 cm, respectively, were maintained. Nitrogen, phosphorus, and potassium (N‐P‐K) were applied at the ratio of 120:60:40 kg ha^−1^, respectively. Nitrogen was applied in three split doses: the first as basal, the second at maximum tillering, and the third at panicle initiation stage. However, P and K were applied as a basal dose. Four days after transplanting, pre‐emergence herbicide Pretilachlor (Syngenta) was broadcasted (1–1.5 L ha^−1^) to control the weeds. Plant protection chemicals and pesticides are used based on the need of the situation in both seasons.

Drought experiment was performed during DS2019 and WS2019, where ILs were planted in augmented randomized complete block design with repeated drought susceptible (IR64) and tolerant check (Sahbhagi dhan) along with the parents. For imposing drought in stress trials during the monsoon season (WS2019; June to September), sowing was delayed for one month (15 July) in WS vs. the NS experiment (15 June).) The rain received during monsoon months June (102 mm), July (80 mm), August (193 mm), and September (294 mm) were normal (ICRISAT weather data). The excess rainwater was removed in case of a spill of rain that happened in between the drought‐imposed period (8, 18, and 28 Sept. 2019). However, in the DS2019, sowing of both the experiments (drought stress and NS) was done at the same time. Water was drained out from the field at 30 d after transplanting in transplanted experiments and irrigation was withheld to impose drought stress cycle at the RS. The stress cycle was continued until severe leaf rolling was observed in Lalat, and water table depth was maintained below 100 cm for >2 wk. Life‐saving irrigation was provided using flash flooding based on the requirement, after which, the second round of the stress was imposed. This process was repeated until maturity. Water table depth was measured by installing the water table tube of 1.10 m unplasticized polyvinyl chloride (U‐PVC) pipe (1 m in below the ground level and 10 cm above ground level) in the experimental fields at regular intervals. Day‐to‐day depletion of the water table was measured using a marked wooden scale.

#### 2.3.1 Observation of yield and yield‐related traits

Yield and yield‐attributing traits, such as data for days to 50% flowering (DFF), plant height (cm), number of the tiller, panicle length (cm), and GY (kg ha^−1^) were recorded for the experiments under stress as well as NS condition. The data of DFF was recorded when 50% of the panicles of the plants of each plot showed exertion. Plant height was measured from the soil surface to the tip of the panicle on the main tiller from three random hills in each plot at the physiological maturity, from which the mean was calculated. The number of tiller and panicle length was recorded before harvesting of the crop. Harvesting was done at physiological maturity after which GY was measured per plot (g). Seeds were dried to 12% moisture before weighing and the plot yield was converted to GY kilogram per hectare (kg ha^–1^).

### 2.4 Phenotypic screening of ILs for BLB resistance

For BLB screening, a highly virulent isolate of *Xanthomonas oryzae* pv. *oryzae* (*Xoo*) (DX‐020) collected from IIRR, Rajendranagar, was used (Yugander et al, 2017). All the F_7_ and F_8_ lines, along with donor, Lalat, and susceptible check (TN1) were screened for their reaction to BLB under field conditions. The bacterial strain was revived on the peptone sucrose agar plate at 25 °C for 2 d and multiplied on peptone sucrose agar media for inoculum production. Bacterial growth was scraped from all the plates and resuspended in sterilized distilled water. The colony‐forming unit (CFU) concentration was adjusted to 10^9^ CFU ml^−1^ by taking the optical density (1.0) at 600 nm using a spectrophotometer. Three plants for each plot were inoculated at a maximum tillering stage with freshly prepared inoculum following the clip inoculation method with sterilized scissors (Kauffman et al., 1973). Five fully expanded leaves per plant were inoculated (2–3 cm from tip) and disease reaction was scored 21 d after inoculation. To reduce the effect of high temperature and to provide favorable conditions for durable entry of bacteria from the clipped site, the inoculation was preferably done in the morning.

The disease assessment was done by measuring the lesion length (cm) from the cut tip of inoculated leaves. The mean of three leaves was taken for determining the reaction type. Disease scoring was done following the IRRI standard evaluation system (SES) scale (IRRI, 1996). A plant was classified as resistant if the average lesion length was 0–3 cm, moderately resistant if the lesion was >3–6 cm, moderately susceptible if the lesion was 6–9 cm, and susceptible if the lesion was longer than 9.0 cm (Chen et al., 2000).

### 2.5 Phenotypic screening of ILs for blast resistance

All the advance generation lines (F_7_ and F_8_) along with donor, Lalat and susceptible check (HR‐12), were evaluated using a highly virulent isolate (SPI‐40) collected from IIRR, Rajendranagar (Madhan Mohan, 2011), under in vivo conditions for their reaction to blast under uniform blast nursery at IRRI–South Asia Hub during DS2019 and WS2019. The seeds of all the lines were planted in a row spacing of 10 cm, which was bordered with two rows of highly susceptible check HR12. Abundant nitrogen (100–120 kg ha^−1^) in the form of urea was applied in two split doses: one‐half at seeding and the other half was applied 15 d after seeding. The blast isolates were cultured and stored on filter paper as described by Hayashi et al (2009). Stored culture colonized on desiccated filter paper was revived and multiplied on oatmeal agar plates for 7 d. For inoculum production, the fungal growth surface was scraped using a sterilized glass slide and filtered through two layers of nylon mesh. The spore concentration was adjusted to 1 × 10^5^ spore ml^−l^. The inoculum was sprayed onto young seedlings at four‐leaf stage using a fine sprayer during which high relative humidity (>90%) was maintained for disease development. One week after inoculation, inoculated seedlings were monitored for the development of blast lesions.

Disease reactions of ILs and parents were recorded 30 d after sowing. Scoring of disease reaction was repeated at 5‐d intervals until >80% of the susceptible checks were infected. Disease reaction was visually scored on a scale of 0 to 9 (SES, IRRI, 1996). A scores of 0–3 was considered resistant, a score of 4 was considered moderately resistant, and scores of 5–9 were considered susceptible.

### 2.6 Evaluation of ILs for grain quality traits

The ILs that showed resistance to blast, BLB, and drought were selected for the analysis of grain quality traits. The selected ILs were grown at International Rice Research Institute, South Asia Regional Centre, Varanasi, which is also were the following grain quality analysis were performed: hulling, milling, head rice recovery (HRR), chalkiness, kernel length (KL), kernel breadth (KB), kernel length/breadth ratio (L/B), alkali spreading value (ASV), and amylose content (AC).

Mature grains of the selected ILs along with Lalat and IR64 (grain quality check) were dried (relative humidity ∼ 12%), dehulled, and polished in a laboratory rice mill (PAZ‐1/DT Zaccaria testing rice mill). A set of 10 grains for each selected line were used to study grain dimension, KL and KB, using image analyzer (Vibe QM3 image analyzer). The L/B of the 10 grains was calculated by dividing mean KL with mean KB. The ASV was estimated with minor modifications as in Little et al. (1958). A group of six polished rice grains from each line was immersed in a freshly prepared 1.7% KOH solution and incubated at 28–30 °C for 23 h; spreading of the rice grains was visually categorized in seven categories from 1 (unaffected) to 7 (completely dissolved). The sample for AC was prepared by the method described by Juliano et al., 1981. The AC was estimated using an automated wet chemistry analyzer (Skalar San^++^), which consists of an autosampler, an amylose chemistry unit.

### 2.7 Statistical analysis

The phenotypic results obtained from drought stress and NS trials have been statistically evaluated using PBTools v1.4 for the measurement of trial mean and standard deviation error. At the 5% significant levels, the LSD was used to evaluate the means of the test entries in addition to which significant differences of the traits were analyzed between parents and each ILs. Broad‐sense heritability (*H* ^2^) and *F* test of each trait were also calculated using PB Tools for understanding the variation.

## 3 RESULTS

### 3.1 Marker‐assisted forward breeding to combine QTL and genes

Multiple QTL and genes in the Lalat background were combined using a simultaneous stepwise crossing scheme. MAFB was employed to combine all QTL and genes, which included genotyping of segregating plants and phenotypic selection to find the best ILs with the most QTL and genes for the traits under study. Parental polymorphism analysis was performed between Lalat and donor parents. Markers that have a base difference of more than 10 bp were selected for use in foreground selection. Simple sequence repeat marker polymorphic at the peak, flanking regions of QTL and gene‐specific or linked marker polymorphic for targeted genes were used for hybridity confirmation and foreground selection (Supplemental Table S1).

In WS2013 single cross of Lalat with four different parents were attempted, that is, Lalat and IR96321‐1447‐561‐B‐1 (*qDTY_1.1_* + *qDTY_3.1_*); Lalat and IR74371‐46‐1‐1 (*qDTY_12.1_*); Lalat and IRBL‐9 (*Pi9*); and Lalat and IRBB‐60 (*Xa4* + *xa5* + *xa13* + *Xa21*); and a total of 90, 121, 96, and 132 F_l_ population seeds were generated (Figure 1). The hybridity of these F_1_ seeds was confirmed with identified polymorphic markers (Supplemental Table S2). All four sets of true F_1_ seeds were crossed to produce two types of intercrosses IC_1_F_1_, that is. Lalat/IR96321‐1447‐561‐B‐1 (*qDTY_1.1_*, *qDTY_3.1_*)//Lalat/IRBB60 (*Xa4* + *xa5* + *xa13* + *Xa21*) and Lalat/IR74371‐46‐1‐1 (*qDTY_12.1_*)//Lalat/IRBL9 (*Pi9*) and 79 and 131 IC_1_F_1_ seeds were produced for the two attempted crosses. The genotypically confirmed IC_1_F_1_ seeds were again intercrossed [Lalat/IR96321‐1447‐561‐B‐1 (*qDTY_1.1_*, *qDTY_3.1_*)//Lalat/IRBB60 (*Xa4* + *xa5* + *xa13* + *Xa21*)] × [Lalat/IR74371‐46‐1‐1 (*qDTY_12.1_*)//Lalat/IRBL9 (*Pi9*)] and a total of 181 IC_2_F_1_ seeds were produced. All IC_2_F_1_ seeds were grown and foreground selection was done with QTL and gene‐specific markers. A total of 680 IC_2_F_2_ plants were grown and genotyped and plants with a maximum of six gene and QTL were found. To combine all the alleles in the single background, two IC_2_F_2_ populations having complementary alleles [IC_2_F_1_ (*qDTY_1.1_*, *qDTY_3.1_*, *Xa4*, *xa5*, *xa13* and *Pi9*) and IC_2_F_1_ (*qDTY_3.1_*, *qDTY_12.1_*, *Xa4*, *xa5*, *Xa21*, and *Pi9*)] were intercrossed (Supplemental Table S3). In the first parent (IC_2_F_2_), the alleles *qDTY3.1*, *xa5*, *xa13*, and *Pi9* were heterozygous and the alleles *qDTY_1.1_* and *Xa4* were homozygous. For the second parent (IC_2_F_2_) alleles *qDTY_3.1_*, *xa5*, *Xa21*, and *Pi9* were heterozygous and the alleles *qDTY_12.1_* and *Xa4* were homozygous. The generated 195 IC_3_F_1_ plants were grown, and three plants with all the targeted genes are selected for further advancement. A total of 1358 IC_3_F_2_ generations were grown, and foreground selection was done. Out of these 13 IC_3_F_2_ plants having all targeted alleles were advanced. At IC_3_F_3_ generation, a total of 5,000 plants were grown and selected plants were genotyped. Nineteen IC_3_F_3_ plants were forwarded as a family, and a total of 1,460 IC_3_F_4_ plants were grown and genotyped. From 1,460 IC_3_F_4_ plants, 28 ILs having five to eight targeted alleles were selected and advanced to IC_3_F_5_. These 28 ILs were also genotyped using an SNP marker (PCR based marker for *xa5*), resulting in the confirmation of ILs with five to seven QTL and genes in homozygous state (Supplemental Table S3). The phenotyping of these ILs for reproductive stage drought tolerance, BLB, and blast resistance was also carried out at F_7_ and F_8_ generations.

### 3.2 Phenotypic performance of ILs under RS drought stress and NS conditions

**FIGURE 2:**
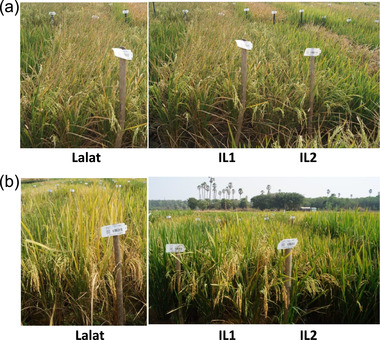
Performance of selected introgression lines (ILs) under reproductive stage drought stress and nonstress condition. (a) Evaluation of selected ILs (IL1, IR 144974‐161‐213‐27‐47‐B, and IL2, IR 144974‐161‐176‐26‐48‐B) and ‘Lalat’ under reproductive stage drought stress condition. (b) Evaluation of selected ILs (IL1, IR 144974‐161‐213‐27‐47‐B, and IL2, IR 144974‐161‐176‐26‐48‐B) and recurrent parents under nonstress conditions

**FIGURE 3:**
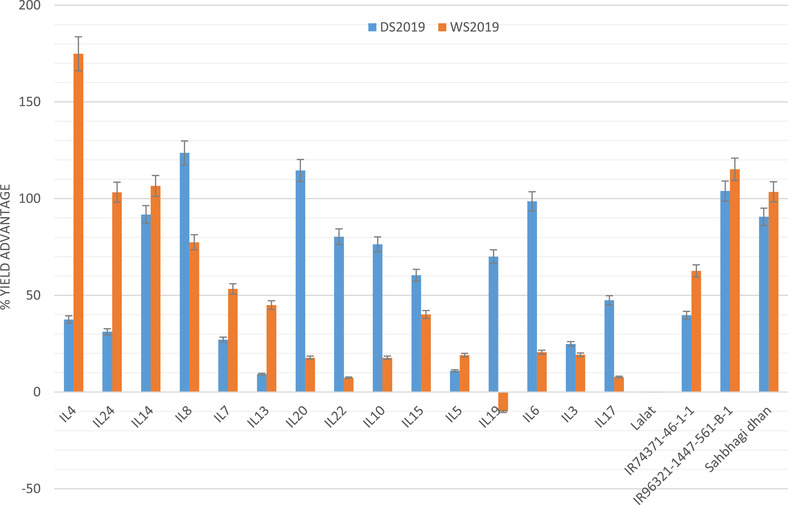
Percentage yield advantage of introgression lines (ILs) over ‘Lalat’ in drought and nonstress condition. Yield advantage of developed ILs along with parents and checks are compared under nonstress and reproductive stage drought condition in both DS2019 and WS2019. IL1, IR 144974‐161‐213‐27‐47‐B; IL2, IR 143207‐134‐476‐146‐51‐57‐8‐B; IL3, IR 143207‐31‐839‐22‐20‐78‐20‐B; IL4, IR 144974‐161‐213‐34‐45‐B; IL5, IR 144974‐161‐176‐28‐49‐B; IL6, IR 144974‐161‐176‐35‐51‐B; IL7, IR 144973‐228‐112‐10‐36‐B; IL8, IR 144973‐228‐112‐2‐37‐B; IL9, IR 143207‐176‐839‐22‐20‐7‐2‐B; IL10, IR 143207‐131‐917‐21‐4‐5‐63‐B; IL11, IR 144974‐161‐213‐24‐41‐B; IL12, IR 143207‐111‐476‐22‐20‐9‐3‐B; IL13, IR 143207‐153‐476‐146‐51‐37‐1‐B; IL14, IR 144974‐161‐213‐22‐44‐B; IL15, IR 144974‐161‐176‐26‐48‐B

The experiment conducted for phenotyping of ILs possessing drought‐tolerant QTL under RS drought and NS conditions during DS2019 and WS2019 resulted in adequate phenotypic differences among the ILs, Lalat, and checks. The results of ANOVA, LSD, means, the heritability of selected ILs with drought tolerant QTL under RS and NS condition of DS2019 and WS2019 are summarized (Supplemental Table S4). During DS2019, severe drought stress (SS) was imposed and the average reduction in yield of ILs was found to be 86.5% vs. the NS condition (Figure 2). The percentage yield advantage of ILs over Lalat varies from 9 to 124% in DS2019 and from 7 to 175% in WS2019 (Figure 3). Under drought stress, 14 ILs have shown a greater yield advantage over Lalat, with the highest yield being 1,120 kg ha^−1^ (92%) and 1,020 kg ha^−1^ (103%) in the DS2019 and WS2019, respectively. IR144974‐161‐213‐27‐47‐B performed very well in WS2019 and yielded 1,413 (175%) kg ha^−1^; however, its yield was only 803 (38%) kg ha^−1^ in DS2019. The least improvement in yield of IR 144974‐331‐112‐12‐33‐B was reported as 601 and 179 kg ha^−1^ in DS2019 and WS2019, respectively. Significant seasonal differences in the yield of a few ILs (IR143207‐153‐476‐146‐51‐37‐1‐B, IR144974‐161‐213‐34‐45‐B, IR143207‐134‐476‐146‐51‐57‐8‐B, and IR144974‐331‐112‐12‐33‐B) were also observed. The trial means of ILs for GY was 587 kg ha^−1^ under SS in DS2019. In WS2019, the average reduction of yield of ILs was 78.5% vs. the NS condition. The trial mean of ILs for GY was 867 kg ha^−1^ under SS in WS2019. Broad‐sense heritability (*H* ^2^) of GY under RS ranged from 0.72 to 0.95 in the years of DS2019 and WS2019. The results of other agronomic parameters under RS drought tolerance showed that the majority of ILs have less reduction in plant height, the number of tillers, and panicle length vs. Lalat. However, the value for all these agronomic parameters of the majority of ILs under NS condition was almost equal to Lalat (Supplemental Table S4).

### 3.3 Assessment for BLB and blast resistance of ILs

**FIGURE 4:**
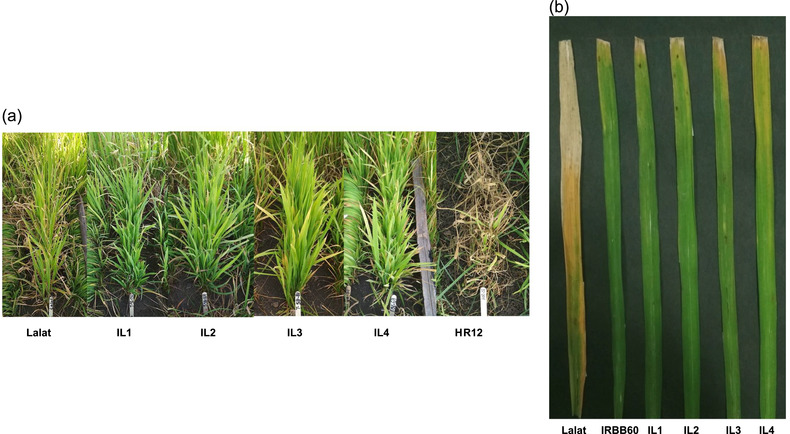
Screening of selected introgression lines (ILs) of ‘Lalat’ against blast and bacterial leaf blight (BLB) diseases. (a) Phenotypic performance of ILs, Lalat susceptible checks under uniform blast nursery. The susceptible check HR12 and Lalat were highly susceptible to blast disease, whereas the tested ILs were found resistant. (b) Phenotypic performance of ILs, recurrent parent, and donor parents. The recurrent parent Lalat was found susceptible, whereas the donor parent (IRBB60) and the selected ILs were found highly resistant to BLB. IL1, IR144974‐161‐213‐27‐47‐B; IL2, IR 144974‐161‐176‐35‐51‐B; IL3, IR143207‐31‐839‐22‐20‐78‐20‐B; IL4, IR144973‐228‐112‐10‐36‐B

Fourteen ILs performed well under both NS and RS drought situations and were evaluated for their reaction to BLB and blast pathogen during DS2019 and WS2019. The reaction of the best six ILs that are resistant against both BLB and blast along with six donors and Lalat are presented in Table 1 and Figure 4. The BLB reaction of the elite parent Lalat and the susceptible check (TN1) expressed susceptible reaction with the average lesion length of 9.7 and 11.3 cm, respectively, in DS2019. However, the reaction of Lalat and TN1 for BLB was 10 and 11.1 cm, respectively, in WS2019. The donor parent IRBB60 possessing *Xa4*, *xa5*, *xa13*, and *Xa21* expressed highly resistant reactions with an average lesion length of 0.75 to 0.54 cm in DS2019 and WS2019, respectively. The list of selected ILs having *Xa4*, *xa5*, *xa13*, and *Xa21* genes in different combinations and have a resistant response to BLB with the average lesion length ranging from 1.3 to 3.0 cm (Table 1, Figure 4). The ILs with *xa5*, *xa13*, and *Xa21* showed a better level (1.3–2.2 cm) of resistance than any other three combinations of genes.

**TABLE 1:** Phenotypic reaction of best introgression lines against blast and bacterial leaf blight (BLB) during DS2019 and WS2019

		**Blast score and reaction** **^b^**				**BLB lesion length and reaction** **^c^**			
		**DS2019**		**WS2019**		**DS2019**		**WS2019**	
**Designation** ^a^	**QTL–gene combination**	**Score**	**Reaction**	**Score**	**Reaction**	**Lesion length**	**Reaction**	**Lesion length**	**Reaction**
						cm		cm	
IR 144974‐161‐213‐27‐47‐B	*qDTY_1.1_*, *qDTY_3.1_*, *qDTY_12.1_*, *Pi9*, *xa5*, *xa13*, *Xa21*	2	R	3	R	2.4 ± 0.37	R	2.88 ± 0.54	R
IR 144974‐161‐176‐35‐51‐B	*qDTY_3.1_*, *qDTY_12.1_*, *Pi9*, *Xa4*, *xa5*, *xa13*, *Xa21*	1	R	2	R	1.3 ± 0.65	R	2.2 ± 0.83	R
IR 143207‐31‐839‐22‐20‐78‐20‐B	*qDTY_1.1_*, *qDTY_3.1_*, *qDTY_12.1_*, *Pi9*, *Xa4*, *Xa21*	3	R	2	R	2.8 ± 0.74	R	3.0 ± 0.86	R
IR 143207‐131‐917‐21‐4‐5‐63‐B	*qDTY_1.1_*, *qDTY_3.1_*, *qDTY_12.1_*, *Pi9*, *Xa4*, *xa5*, *Xa21*	2	R	3	R	2.33 ± 0.53	R	2.46 ± 0.28	R
IR 144974‐161‐176‐26‐48‐B	*qDTY_3.1_*, *qDTY_12.1_*, *Pi9*, *Xa4*, *xa5*, *Xa21*	3	R	3	R	2.8 ± 0.83	R	2.32 ± 0.45	R
IR 144973‐228‐112‐10‐36‐B	*qDTY_1.1_*, *qDTY_3.1_*, *qDTY_12.1_*, *Pi9*, *Xa4*, *xa5*, *Xa21*	2	R	3	R	2.6 ± 0.83	R	2.8 ± 0.65	R
‘Lalat’	–	6	S	6	S	9.7 ± 0.48	S	10.0 ± 0.67	S
IRBL9	*Pi9*	2	R	1	R	–	–	–	–
HR12	–	8	S	7	S	–	–	–	–
IRBB60	*Xa4*, *xa5*, *xa13*, *Xa21*	–	–	–	–	0.75 ± 0.26	R	0.54 ± 0.31	R
TN1	–	–	–	–	–	11.3 ± 0.48	S	10.1 ± 0.72	S

^a^ HR12, blast susceptible check; IRBL9, blast donor; TN1, BLB susceptible check; IRBB60, BLB donor parent.

^b^ Introgression lines expressed blast score 0–3 are considered R (resistant), 4 as MR (moderately resistant), and 5–9 as S (susceptible).

^c^ BLB screening, introgression lines showing mean lesion length (mean ± SD) 0–3 cm as R (resistant), >3–6 as MR (moderately resistant), and >6–9 as S (susceptible).

The blast reaction of donor parent IRBL9 expressed resistant reaction with SES scores 1–2, Lalat expressed susceptible score 6, susceptible check HR12 expressed highly susceptible reaction with SES score 7–8. All the ILs expressed resistance reactions with SES score 0–3 in both the seasons (Table 1, Figure 4). The reaction of ILs indicated that the pyramiding of BLB‐ and blast‐resistant genes in the elite parent Lalat imparted a high level of resistance as indicated by reduced lesion length for BLB disease and resistant score for blast disease.

### 3.4 Grain quality analysis of promising ILs

Fourteen ILs selected based on phenotypic performance under RS and NS conditions were subjected to grain quality analysis, which included physical and cooking quality parameters such as hulling percentage, milling percentage, HRR, KL, KB, L/B, chalkiness, ASV, and AC. Of the 14 ILs selected for analysis, four entries are rejected because of their chalkiness property. From the 10 ILs analyzed, six of the ILs had grain quality characteristics mostly similar to Lalat and IR64, and these ILs also show BLB and blast resistance (Table 2). Grain quality value of six ILs range as follows: hulling percentage (80.31–81.82%), milling percentage (72.43–73.39%), KL (6.35–7.01 mm), KB (2.13–2.28 mm), and L/B (2.98–3.20). Also, we observed that the grain physical characteristics of these ILs were almost similar to elite parent Lalat and grain quality check IR64 (Table 2). Head rice recovery of ILs varied from 65.51 to 67.96%, which is similar to Lalat (HRR 64.90%). The AC of the selected ILs ranged from 23.16 to 25.77% (intermediate), which was similar to Lalat (24.94%) and IR64 (22.38%). The ASV of all six ILs was 5, which was similar to the ASV of Lalat and IR64. Also, the grain type of all the selected ILs was long slender and almost identical to Lalat.

**TABLE 2:** Grain quality analysis of promising introgression lines

	**Grain yield**										
**Designation**	**DS 2019**	**WS2019**	**Hulling**	**Milling**	**Head rice recovery**	**Kernel length**	**Kernel breadth**	**Length/breadth ratio**	**Kernel type**	**Alkali spreading value**	**Amylose content**
	kg ha^−1^		%			mm					%
IR 144974‐161‐213‐27‐47‐B	5,383	4,194	80.31	72.43	66.89	6.35	2.13	2.98	LS^a^	5	24.36
IR 144974‐161‐176‐35‐51‐B	4,417	4,595	80.71	72.95	65.51	6.87	2.25	3.06	LS	5	25.77
IR 143207‐31‐839‐22‐20‐78‐20‐B	4,375	4,384	80.91	73.01	67.96	7.01	2.28	3.07	LS	5	24.39
IR 143207‐131‐917‐21‐4‐5‐63‐B	4,250	5,084	80.74	72.61	68.39	7.10	2.29	3.10	LS	5	24.79
IR 144974‐161‐176‐26‐48‐B	4,833	5,873	80.91	73.01	67.96	7.01	2.28	3.07	LS	5	24.39
IR 144973‐228‐112‐10‐36‐B	4,750	4,022	81.82	73.39	66.53	6.80	2.13	3.20	LS	5	23.16
‘Lalat’	3,591	4,364	81.02	72.92	64.90	7.04	2.22	3.17	LS	5	24.94
‘IR64’^b^	–	–	79.64	71.40	59.85	7.01	2.13	3.30	LS	5	22.38

^a^ LS, long slender.

^b^ IR64 was used as grain quality check.

## 4 DISCUSSION

Over the years, we have observed that most of the rice growing areas face severe abiotic and biotic stresses resulting in reduced yield and poor grain quality (Das & Rao, 2015). In the global context of crop loss because of stresses and its resulting upsurge in the hunger strike, developing high‐yielding, climate‐resilient, and superior grain quality rice using modern breeding approaches is a sustainable way to ensure food security in rice cultivation. In this study, we deployed a crossing strategy with previously identified QTL and closely linked or gene‐based markers to combine multiple stress resistance into elite rice cultivar Lalat and validate the tolerance or resistance by phenotyping for respective stresses using standard screening protocol (IRRI, 2013).

The simultaneous crossing program for combining all the targeted QTL and genes for drought, BLB, and blast has resulted in combining all the targeted traits in one root genotype. First, all four donors (IR96321‐1447‐561‐B‐1; IR74371‐46‐1‐1; IRBB60; and IRBL9) were crossed with elite parent Lalat, then the developed F_1_ plants were intercrossed and double crossed to segregate all the QTL and genes combined. All the donors were crossed with elite parents in a way to capture the majority of the traits of the elite parent type, while MAFB was used for quick delivery of the finished product by escaping two or more backcrosses needed for the MABB approach. The result of stacking multiple QTL and genes in one step indicates the use of a simultaneous crossing program coupled with MAFB could be an effective breeding approach using the elite donors used in the crossing program. During the advancement of complex IC_3_F_2_ generations, both genotypic and visual selections were practiced (plant type, grain type, DFF) that also involved selection of lines with different combinations of QTL and genes including some lines that lacked one or two QTL and genes.

Complex IC_2_F_1_ plants (181) have been used to grow F_2_ populations, keeping in mind to find the best recombinants for yield, plant type, and grain type with all possible combinations of QTL and genes. While we could not achieve all the targeted alleles at IC_2_ stage, we again attempted intercross between IC_2_F_2_ plants having complementary alleles (Supplemental Table S3) and three IC_3_F_1_ plants with all the targeted alleles were found. The segregating population was maintained as 1,358 plants in IC_3_F_2_, 5,000 in IC_3_F_3_, 1,460 in IC_3_F_4_, 28 families in IC_3_F_5_, and IC_3_F_6_ generations have been used for selective genotyping as well as phenotyping at field level. The selection strategy adapted in the present breeding program is practical, where plants from segregating generations were selected based on both genotyping (foreground markers) as well as the phenotypic selection at field level for plant type and grain type from segregation populations. The ILs were selected in the field by comparing the phenotypic expression with respective parents or checks and grain type with Lalat.

In the process of development of multiple stress‐tolerant lines, three QTL for GY under drought (*qDTY_1.1_*, *qDTY_3.1_*, and *qDTY_12.1_*) that gives yield advantage of 800–1,000 kg h^−1^ in combination (Bernier et al., 2007; Ghimire et al., 2012; Kumar et al., 2008; Venuprasad et al., 2009; Vikram et al., 2011) has been targeted for introgression in the current breeding program for Lalat. Previously, the transfer of drought‐tolerant QTL, that is, *qDTY_1.1_*, *qDTY_1.2_*, *qDTY_2.1_*, *qDTY_2.2_*, *qDTY_2.3_*, *qDTY_3.1_*, *qDTY_3.2_*, and *qDTY_12.1_*, have been carried out extensively using MABB approach, which has resulted in a drought‐tolerant version of some of the mega rice cultivars such as ‘Swarna’ (*qDTY_1.1_* + *qDTY_2.1_* + *qDTY_3.1_* + *Sub1*) as ‘CR dhan 801’, Swarna (*qDTY_2.1_* + *qDTY_3.1_* + *Sub1*) as ‘CR dhan 802’, IR64 (*qDTY_2.2_*, *qDTY_4.1_*) as ‘DRR dhan 42’, and ‘Samba Mahsuri’ (*qDTY_2.1_* + *qDTY_3.1_* + *Sub1*) as ‘DRR dhan 50’ have been released as cultivars in various countries of southern Asia for cultivation (Kumar et al., 2014, 2018; Sandhu et al., 2019; Bhandari et al., 2019). In our previous reports, 51% of the total genetic variance with an estimated additive effect of 172 kg ha^−1^ for yield was observed under upland RS over 2 yr of field evaluation through the introgression of *qDTY_12.1_* (Bernier et al., 2009). Drought tolerant QTL *qDTY_1.1_* and *qDTY_3.1_* have yield advantage of 72–1,600 kg ha^−1^ over Swarna under RS drought (Sandhu et al., 2019).

In the current study, ILs with drought‐tolerant QTL were tested under RS drought tolerance in DS2019 and WS2019, respectively, which resulted in adequate yield improvement over Lalat (IR 144974‐161‐213‐27‐47‐B, IR 144974‐161‐176‐35‐51‐B, IR 143207‐31‐839‐22‐20‐78‐20‐B, IR 143207‐131‐917‐21‐4‐5‐63‐B, IR 144974‐161‐176‐26‐48‐B, and IR 144973‐228‐112‐10‐36‐B), where yield was found to be comparable with drought check ‘Sahbhagi dhan’. These ILs showed less reduction in yield under drought than recurrent parent Lalat. In both seasons, ILs (IR 144974‐161‐213‐27‐47‐B, IR 143207‐31‐839‐22‐20‐78‐20‐B, IR 144973‐228‐112‐10‐36‐B, IR 144973‐228‐112‐2‐37‐B, and IR 143207‐134‐476‐146‐51‐57‐8‐B) with all three drought‐tolerant QTL (*qDTY_1.1_*, *qDTY_3.1_*, and *qDTY_12.1_*) gave a greater yield advantage under drought over elite parent Lalat. In DS2019, the percentage GY advantage of ILs against Lalat varies from 9 to 124%, while in WS2019, it ranges from 7 to 175% (Figure 3). Like the result of this study, yield advantage of 1,200 to 2,000 kg ha^−1^ under RS have been reported after introgression of two to three *qDTY* in IR64 background (Swamy et al., 2013). Pyramided lines developed using three drought‐tolerant QTL (*qDTY_1.1_*, *qDTY_2.1_*, *qDTY_3.1_*) showed yield advantage of 200–1,700 kg ha^−1^ under RS drought stress in Swarna background (Sandhu et al., 2019).

In the context of biotic stresses, three BLB resistance genes (*xa5*, *xa13*, and *Xa21*) from IRBB60 were transferred to elite parent Lalat for providing durable resistance against BLB through MAFB. Lalat itself has the *Xa4* gene in the background but is susceptible to almost all the isolates, which suggests its breakdown (Dokku et al., 2013b). Pyramiding of multiple BLB‐resistant genes is a prerequisite because of the existence of numerous genetically distinct virulent *Xoo* strains prevailing in diverse rice growing areas in India. Deployment of combination of genes can provide broad‐spectrum, durable resistance in BLB‐prone rice growing areas (Dokku et al., 2013a; Singh et al., 2001; Suh et al., 2011; Pradhan et al., 2015). The ILs with *xa5* + *xa13* + *Xa21* genes showed higher resistance than ILs with *Xa4* + *xa5* + *Xa21* gene combinations. However, ILs with all four genes (*Xa4*, *xa5*, *xa13*, and *Xa21*) was the most effective combination against BLB isolates (lesion length, 1.3–2.2 cm) when compared with any of two combinations. The current results validate the importance of gene pyramiding and the selection of effective genes in the breeding program. The ILs with two genes in different combinations showed different levels of resistance. Similar resistance responses were also reported by a few other studies in rice, for example, Yoshimura et al. (1995) reported that rice lines carrying *Xa4* + *xa5* genes were more resistant to race of isolate 4 than lines carrying any single gene, parents, or combination of *Xa4* + *Xa10* genes. Breeding lines developed with two, three, and four BLB resistance genes show a wide spectrum of disease response. However, lines with either *xa5* + *xa13* + *Xa21* and *Xa4* + *xa5* + *xa13* + *Xa21* genes have higher levels of disease resistance than any other combination. Rice variety PR106 pyramided with a combination of *xa5*, *xa13*, and *Xa21* genes has a differential level of resistance. Lines with either *Xa21* or lines with *Xa21*, *xa13*, and xa5 genes have resistant responses against all tested races of Punjab and Philippines (Huang et al., 1997). The ILs developed with *xa5*, *xa13*, and *Xa21* genes in the rice cultivar Triguna have differential resistance response. The ILs carrying the two‐gene combinations *Xa21* and *Xa13* were found to exhibit higher levels of resistance against disease than any other two gene combinations (Sundaram et al., 2009). Dokku et a. (2013a) pyramided *Xa4*, *xa5*, *xa13*, and *Xa21* genes in rice, and lines with different gene combinations were developed. The performance of lines with *Xa4* + *xa13* + *Xa21* genes were better than other three‐gene combinations (*Xa4* + *xa5* + *Xa21* and *Xa4* + *xa5* + *xa13*) as well as all the two‐gene combinations like *Xa4* + *xa5*, *Xa4* + *xa13*, or *Xa4* + *Xa21*.

In the context of blast disease, a single, broad‐spectrum, blast‐resistant gene, *Pi9*, has been deployed in current study using MAFB. Though several resistant genes for blast have been identified, *Pi9* was found to offer broad‐spectrum resistance against blast. In our study, ILs with *Pi9* showed a good resistance score (1–3) in both the seasons. Similarly, Wen & Gao (2012) introgressed *Pi‐9(t)* into hybrid restorer line Luhui17 by using MABB, and a higher level of resistance was observed. Khanna et al (2015) introgressed two blast‐resistant genes, *Pi9* and *Pi2*, in ‘Pusa Basmati 1’ and found that ILs carrying *Pi9* are resistant against all three isolates, while ILs carrying *Pi2* was resistant against only one isolate. The study observed that the use of single, broad‐spectrum blast resistance genes could be effective against blast disease.

In the present study, in addition to the increased level of drought tolerance, BLB and blast resistance stringent selection was followed to develop ILs that are agronomically similar or superior to Lalat. Lalat has long slender grain type in comparison with the medium bold donor used for drought tolerance, BLB, and blast. However, with stringent phenotypic selection based on plant morphology and grain characteristics at the early generation, we selected plants that were almost like Lalat for grain appearance (length, breadth). The ILs with different grain appearance were also generated but not included for further advancement in this study. As a result, all the selected ILs were equivalent to Lalat for most of the grain quality characteristics. A good level of similarity in grain quality traits of ILs, compared with Lalat, was achieved in this study. Development of such ILs with multiple QTL and genes for various abiotic and biotic stress tolerance traits will certainly help farmers to harvest higher yield under the occurrence of drought, BLB, and blast when they occur alone or in combinations.

The breeding strategy blend with use of robust molecular markers available for the targeted traits in the present study had resulted in accumulating favorable alleles more precisely, quickly, and effectively, particularly in pyramiding multiple complex traits and hence can maximize the expected genetic gain targeted in rice to feed the ever‐growing population under the ongoing adverse climatic changes. The breeding strategies adopted in the present study facilitated the development of new rice lines with higher yield with good grain quality in less than 5 yr, which enhanced the selection efficiency that led to faster development of rice cultivars. This is one of the few studies targeted to combine tolerance or resistance to both biotic and abiotic stresses using MAFB.

The novel feature of this study was a successful demonstration of multiple QTL and genes in a single genetic background through a unique crossing program more particularly using phenotypic selection strategy together with genotyping. The plant selection practiced in the study via morphological and grain quality traits to enhance the recovery of the recurrent parent genome was successful, and plants with elite parent phenotype and grain type were developed. Common genetic background from elite parents in each intercross coupled with reliable phenotyping and screening at field level enabled us to reject plant types differing from Lalat, so only those ILs resembling Lalat in maximum essential morphological and quality features were considered for next advancement. This strategy helped us to move ahead without need of background genotyping. Background genotyping is more useful in backcross breeding when the chances of obtaining a recurrent type are higher, whereas in MABF, where many parents are involved, visual selection from a larger number of lines is useful. This is one of the few studies where we have not carried out background genotyping at any generation. This study is in agreement with the previous studies that used morphological and quality‐based plant selection strategies in selecting breeding lines (Das et al., 2018; Kumar et al., 2018). The stress‐tolerant lines developed in the current study can be released as a cultivar through a national varietal release system or can be used as a donor parent in performing elite–elite crosses to combine biotic and abiotic stresses tolerance or resistance.

The study concludes that a stepwise crossing scheme coupled with MAFB works well for combining multiple QTL and genes governing multiple abiotic and biotic stresses. Maintenance of many plants and progenies at the F_1_ stage and early generations enabled us to get all combinations of QTL and genes with better plant types. The ILs with all three drought‐tolerant QTL (*qDTY_1.1_*, *qDTY_3.1_*, and *qDTY_12.1_*) provided higher yield under RS drought. The ILs with *Xa4*, *xa5*, *xa13*, and *Xa21* genes showed higher levels of resistance against BLB followed by *xa13* + *Xa21* and *xa5* + *Xa21*. The ILs developed with blast gene (*Pi9*) showed high to medium resistance score. The grain physical quality and cooking quality is almost similar to elite parent Lalat. The promising ILs (IR144974‐161‐213‐27‐47‐B; IR144974‐161‐176‐35‐51‐B; IR143207‐31‐839‐22‐20‐78‐20‐B; IR143207‐131‐917‐21‐4‐5‐63‐B; IR144974‐161‐176‐26‐48‐B; and IR144973‐228‐112‐10‐36‐B) with tolerance to drought and resistance to BLB and blast together with good grain quality traits has been promoted for evaluation in national trials. Availability of these ILs possessing genes and QTL in elite background provides an opportunity to make MAFB less cumbersome, quicker, as well as enable us to combine higher number of genes governing tolerance to multiple abiotic and biotic stresses with less worry for undesirable linkages, which is a major limitation while using traditional donors in MABB or marker‐assisted selection programs.

## AUTHOR CONTRIBUTIONS

Uma Maheshwar Singh: Data curation, Formal analysis, Investigation, Methodology, Writing‐original draft, Writing‐review & editing. Shilpi Dixit: Data curation, Formal analysis, Methodology, Writing‐review & editing. Shamshad Alam: Data curation, Formal analysis, Investigation, Methodology, Writing‐review & editing. Shailesh Yadav: Formal analysis, Investigation, Methodology, Writing‐review & editing. Vinukonda Vishnu Prasanth: Data curation, Methodology. Arun Kumar Singh: Data curation, Investigation, Methodology. Challa Venkateshwarlu: Formal analysis, Methodology. Ragavendran Abbai: Formal analysis, Software, Visualization, Writing‐review & editing. Abhilash Kumar Vipparla: Writing‐review & editing. Jyothi Badri: Data curation, Methodology, Resources, Visualization. Tilatoo Ram: Conceptualization, Data curation, Methodology. Madamshetty Srinivas Prasad: Data curation. Gouri Sankar Laha: Data curation. Vikas Kumar Singh: Conceptualization, Project administration, Resources, Supervision, Validation, Writing‐review & editing. Arvind Kumar: Conceptualization, Investigation, Project administration, Resources, Supervision, Validation, Writing‐review & editing.

## CONFLICT OF INTEREST

The authors do not have any conflicts of interest.

## Supporting information


**Supplemental Table S1**. Details of QTL and genes associated markers used for foreground selection. SSR marker from peak and flanking position of drought tolerant QTLs (*qDTY_1.1_*, *qDTY_3.1_*, *qDTY_12.1_*) were used, while gene specific markers for *xa5*, *xa13*, *Xa21*, *Pi9* and gene linked marker for *Xa4* gene are used.

**Supplemental Table S2**. List of primers used for foreground selection. The finally selected polymorphic marker primer sequence is given here.

**Supplemental Table S3**. List of early generation genotyping information. The genotyping details of IC_2_F_2_ and IC_3_F_6_ lines are given. H‐heterozygous, HM‐homozygous and NG‐ negative

**Supplemental Table S4**. ANOVA and mean performance of the introgression lines. The analysis agronomic data of 28 ILs are presented for DS2019 and WS2019 season.

## Data Availability

The relevant supplemental data has been provided with the manuscript. The developed introgression lines can be obtained from IRRI with proper material‐transfer agreement (MTA) or a standard material transfer agreement (SMTA).
