# Supplementary material for: Marker‐assisted forward breeding to develop a drought‐, bacterial‐leaf‐blight‐, and blast‐resistant rice cultivar
Source: Plant Genome. 2021 Nov 29;15(1):e20170. doi: 10.1002/tpg2.20170 (PMC12807386; doi:10.1002/tpg2.20170)
Supplement: Supplementary file 1 [file TPG2-15-e20170-s001.docx]

**Table S2.** List of primers used for foreground selection

| **Target traits** | **Link gene/QTLs** | **Markers** | **Chr** | **Primer sequence (Forward & Reverse)** | **Reference** |
| --- | --- | --- | --- | --- | --- |
| BLB | *Xa4* | *Xa4 gene* | 11 | ATCGATCGATCTTCACGAGG | Ma et al.,1999 |
|  |  |  |  | TGCTATAAAAGGCATTCGG |  |
|  | *xa5* | *Xa5DRR* | 5 | TAGCTGCTGCCGTGCTGTGC | Iyer and McCouch, 2004 |
|  |  |  |  | AATATTTCAGTGTGCATCTC |  |
|  | *xa13* | *Xa13prom* | 8 | GGCCATGGCTCAGTGTTTAT | Chu et al,  2006 |
|  |  |  |  | GAGCTCCAGCTCTCCAAATG |  |
|  | *Xa21* | *pTA248* | 11 | AGACGCGGAAGGGTGGTTCCCGGA | Ronald et al.,1992 |
|  |  |  |  | AGACCGGTAATCGAAAGATGAAA |  |
| Blast | *Pi9* | *Pi9STS2* | 6 | TGCTGACTGCTTGCTATTCGT | Qu et al.,2006; Rathaur et al., 2016 |
|  |  |  |  | GTACTTCATCTTGAGCGACGCAA |  |
| Drought | *qDTY_1.1_* | RM431 | 1 | T GCTGCTTGCCTGCTTCCTTT | Vikram et al., 2011 |
|  |  |  |  | GAAACGAATCAATCCACGGC |  |
|  | *qDTY_3.1_* | RM168 | 3 | TCCTGCGAACTGAAGAGTTG | Venuprasad et al 2009 |
|  |  |  |  | GAGCAAAACCCTGGTTCAC |  |
|  | *qDTY_12.1_* | RM511 | 12 | AACGAAAGCGAAGCTGTCTCC | Mishra et al 2013 |
|  |  |  |  | ATTTGTTCCCTTCCTTCGATCC |  |
