# Supplementary material for: Marker‐assisted forward breeding to develop a drought‐, bacterial‐leaf‐blight‐, and blast‐resistant rice cultivar
Source: Plant Genome. 2021 Nov 29;15(1):e20170. doi: 10.1002/tpg2.20170 (PMC12807386; doi:10.1002/tpg2.20170)
Supplement: Supplementary file 2 [file TPG2-15-e20170-s002.docx]

**Table S1.** Details of QTLs/genes associated markers used for foreground selection

| **Trait** | **Donors** | **QTLs /genes** | **SSR/Gene specific marker screened** | **Markers selected** |
| --- | --- | --- | --- | --- |
| Drought | IR74371-46-1-1 | *qDTY_12.1_* | RM511, RM28166, RM28199, RM1261, RM28099, RM28089 | RM28089 (LFM) |
|  |  |  |  | RM511/RM28166 (PM) |
|  |  |  |  | RM28199 (RFM) |
|  |  |  |  |  |
|  | IR96321-1447 | *qDTY_1.1_* | RM11943, RM212, RM315, RM3825, RM12091, RM12023, RM431, RM11941, RM212, RM315, RM3825 | RM3825(LFM) |
|  | -561-B-1 |  |  | RM431 (PM) |
|  |  |  |  | RM12091 (RFM) |
|  |  | *qDTY_3.1_* | RM15736, RM15738, RM15926, RM15924, RM16032, RM15914, RM15925, RM55, RM168, RM186, RM293, RM468, RM571, RM16030,RM16011, RM16049, RM416, RM520 | RM168 (LFM) |
|  |  |  |  |  |
|  | IR96321-1447 |  |  | -- |
|  | -561-B-1 |  |  | RM16011 (RFM) |
| BLB | IRBB60 | *Xa4* | *Xa4*, RM27317, RM27318, RM27328, RM27333, RM27336, RM27340, RM27354, RM27356, RM27322, RM5538, RM7228, RM114, RM224, RM277, RM201, RM7654 | *Xa4* |
|  |  | *xa5* | xa5DRR, RM122, RM159, RM13 | *xa5*DRR |
|  |  | *xa13* | *xa13* P | *xa13* P |
|  |  | *Xa21* | *Xa21* | *pTA248* |
| Blast | IRBL9 | *Pi9* | *Pi9*STS1F, *Pi9*STS2F, *Pi9*STS3F, *Pi9*, RM527, RM3330, RM12705, RM19835 | *Pi9*STS2 |

LFM: left flanking marker; PM: peak marker; RFM: right flanking marker
