# Supplementary material for: Marker‐assisted forward breeding to develop a drought‐, bacterial‐leaf‐blight‐, and blast‐resistant rice cultivar
Source: Plant Genome. 2021 Nov 29;15(1):e20170. doi: 10.1002/tpg2.20170 (PMC12807386; doi:10.1002/tpg2.20170)
Supplement: Supplementary file 3 [file TPG2-15-e20170-s003.docx]

**Table S4. Mean agronomic performance of selected introgression line under non-stress and reproductive stage drought stress condition**

| **Trait** | **DFF** | | | | **PHT** | | | | **NT** | | | | **PL** | | | | **GYKGPH** | | | | |
| --- | --- | --- | --- | --- | --- | --- | --- | --- | --- | --- | --- | --- | --- | --- | --- | --- | --- | --- | --- | --- | --- |
| **Season** | **DS2019** | | **WS2019** | | **DS2019** | | **WS2019** | | **DS2019** | | **WS2019** | | **DS2019** | | **WS2019** | | **DS2019** | | | **WS2019** | |
| **Targeted**  **condition** | **NS** | **RS** | **NS** | **RS** | **NS** | **RS** | **NS** | **RS** | **NS** | **RS** | **NS** | **RS** | **NS** | **RS** | **NS** | **RS** | **NS** | **RS** | **NS** | | **RS** |
| IR 144974-161-176-33-52-B | 100 | 103 | 94 | 103 | 105 | 88 | 84 | 63 | 7 | 9 | 13 | 11 | 26 | 24 | 23 | 21 | 3708 | 746 | 4944 | | 280 |
| IR 144974-161-213-29-43-B | 87 | 106 | 91 | 112 | 103 | 91 | 80 | 59 | 8 | 7 | 10 | 10 | 27 | 21 | 22 | 19 | 3083 | 708 | 4383 | | 230 |
| IR 144974-161-213-22-44-B | 96 | 98 | 93 | 106 | 100 | 102 | 81 | 58 | 10 | 9 | 14 | 10 | 26 | 22 | 22 | 21 | 3583 | 729 | 5455 | | 613 |
| IR 144974-161-213-27-47-B | 87 | 99 | 90 | 99 | 115 | 103 | 86 | 69 | 12 | 11 | 8 | 7 | 25 | 24 | 22 | 23 | 5383 | 803 | 4194 | | 1413 |
| IR 144974-161-213-24-41-B | 87 | 95 | 88 | 97 | 99 | 94 | 77 | 55 | 8 | 8 | 9 | 8 | 24 | 22 | 21 | 18 | 3363 | 648 | 4467 | | 612 |
| IR 143207-153-476-146-51-37-1-B | 87 | 98 | 84 | 100 | 102 | 92 | 78 | 60 | 8 | 7 | 12 | 11 | 26 | 25 | 22 | 19 | 5542 | 1160 | 4962 | | 620 |
| IR 144974-161-176-28-49-B | 85 | 94 | 90 | 99 | 94 | 94 | 76 | 63 | 9 | 6 | 10 | 9 | 25 | 22 | 23 | 22 | 3750 | 742 | 4322 | | 788 |
| IR 144974-161-213-34-45-B | 86 | 99 | 91 | 99 | 95 | 99 | 78 | 59 | 9 | 8 | 13 | 7 | 26 | 24 | 23 | 20 | 3254 | 1306 | 4345 | | 912 |
| IR 144974-331-112-8-35-B | 87 | 96 | 90 | 99 | 94 | 93 | 80 | 61 | 8 | 8 | 11 | 8 | 24 | 23 | 22 | 22 | 3500 | 881 | 5005 | | 355 |
| IR 143207-176-839-22-20-7-2-B | 80 | 93 | 91 | 116 | 115 | 97 | 77 | 58 | 11 | 11 | 9 | 8 | 27 | 25 | 20 | 19 | 4167 | 1030 | 4255 | | 605 |
| IR 144974-161-213-24-42-B | 97 | 108 | 90 | 99 | 104 | 88 | 80 | 60 | 9 | 8 | 11 | 9 | 26 | 22 | 20 | 20 | 3392 | 912 | 4010 | | 363 |
| IR 143207-158-1178-45-4-29-68-B | 83 | 105 | 92 | 119 | 107 | 104 | 80 | 63 | 9 | 7 | 10 | 8 | 26 | 24 | 22 | 21 | 4317 | 1037 | 3360 | | 421 |
| IR 144974-161-176-35-51-B | 87 | 96 | 91 | 101 | 101 | 88 | 82 | 61 | 6 | 7 | 13 | 11 | 24 | 23 | 23 | 20 | 4417 | 638 | 4595 | | 745 |
| IR 143207-31-839-22-20-78-20-B | 84 | 100 | 86 | 96 | 98 | 84 | 77 | 54 | 9 | 7 | 13 | 10 | 26 | 24 | 23 | 17 | 4375 | 1120 | 4384 | | 1062 |
| IR 143207-131-917-21-4-5-63-B | 80 | 94 | 93 | 103 | 99 | 85 | 74 | 57 | 10 | 7 | 11 | 10 | 23 | 25 | 21 | 19 | 4250 | 937 | 5084 | | 720 |
| IR 144974-331-112-23-39-B | 86 | 101 | 94 | 100 | 99 | 93 | 78 | 59 | 12 | 9 | 10 | 8 | 27 | 25 | 22 | 19 | 5242 | 969 | 5728 | | 412 |
| IR 144974-161-176-26-48-B | 93 | 109 | 91 | 99 | 95 | 92 | 75 | 54 | 9 | 9 | 13 | 7 | 25 | 25 | 21 | 19 | 4833 | 861 | 5873 | | 554 |
| IR 144974-331-112-3-38-B | 100 | 116 | 99 | 120 | 102 | 87 | 72 | 70 | 10 | 8 | 11 | 9 | 26 | 24 | 22 | 21 | 4583 | 870 | 5266 | | 221 |
| IR 143207-111-476-22-20-9-3-B | 77 | 98 | 91 | 98 | 98 | 100 | 72 | 55 | 13 | 11 | 11 | 10 | 25 | 25 | 21 | 18 | 4083 | 993 | 3684 | | 462 |
| IR 144973-228-112-10-36-B | 90 | 103 | 95 | 106 | 95 | 85 | 83 | 64 | 8 | 8 | 10 | 7 | 27 | 25 | 22 | 20 | 4750 | 1253 | 4022 | | 605 |
| IR 144972-376-112-5-32-B | 86 | 97 | 91 | 101 | 91 | 85 | 74 | 58 | 10 | 8 | 11 | 9 | 24 | 23 | 22 | 20 | 3408 | 377 | 4662 | | 529 |
| IR 144973-228-112-2-37-B | 73 | 108 | 84 | 121 | 100 | 106 | 73 | 58 | 9 | 9 | 12 | 10 | 26 | 26 | 20 | 17 | 3292 | 1053 | 3723 | | 552 |
| IR 144974-161-213-25-46-B | 88 | 97 | 90 | 97 | 96 | 98 | 71 | 52 | 7 | 8 | 12 | 9 | 26 | 24 | 20 | 16 | 3458 | 888 | 3184 | | 329 |
| IR 143207-134-476-146-51-57-8-B | 80 | 95 | 86 | 99 | 87 | 81 | 74 | 61 | 8 | 8 | 11 | 9 | 24 | 21 | 20 | 17 | 3333 | 766 | 3728 | | 1045 |
| IR 144974-331-112-12-33-B | 90 | 102 | 88 | 99 | 101 | 81 | 84 | 58 | 8 | 9 | 11 | 10 | 24 | 21 | 23 | 19 | 2917 | 601 | 3584 | | 179 |
| IR 144974-331-112-11-40-B | 90 | 106 | 84 | 101 | 102 | 78 | 68 | 55 | 9 | 9 | 12 | 10 | 26 | 22 | 21 | 19 | 3175 | 495 | 3516 | | 230 |
| IR 144974-331-112-6-34-B | 89 | 118 | 85 | 119 | 94 | 79 | 67 | 57 | 10 | 10 | 11 | 6 | 24 | 20 | 19 | 18 | 4708 | 785 | 3005 | | 246 |
| IR 144974-331-112-18-31-B | 89 | 109 | 82 | 99 | 93 | 76 | 69 | 55 | 11 | 9 | 14 | 9 | 25 | 20 | 20 | 19 | 4263 | 729 | 2866 | | 184 |
| IR74371-46-1-1 | 88 | 111 | 89 | 116 | 102 | 93 | 96 | 82 | 8 | 7 | 11 | 9 | 25 | 24 | 24 | 22 | 4050 | 816 | 4236 | | 836 |
| IR96321-1447-561-B-1 | 86 | 110 | 96 | 120 | 97 | 92 | 74 | 63 | 10 | 9 | 12 | 10 | 25 | 24 | 23 | 22 | 4299 | 1191 | 4988 | | 1106 |
| Lalat | 88 | 100 | 87 | 100 | 110 | 80 | 85 | 66 | 10 | 4 | 11 | 7 | 27 | 23 | 23 | 21 | 3591 | 584 | 4364 | | 514 |
| Sahabagi dhan | 86 | 96 | 88 | 102 | 111 | 105 | 92 | 71 | 9 | 8 | 10 | 9 | 26 | 24 | 23 | 22 | 5439 | 1113 | 5292 | | 1046 |
| Trial mean | 87 | 102 | 90 | 104 | 100 | 91 | 78 | 61 | 9 | 8 | 11 | 9 | 25 | 23 | 22 | 20 | 4047 | 867 | 4359 | | 587 |
| F value | 12 | 2 | 4 | 8 | 8 | 22 | 7 | 7 | 7 | 4 | 1 | 1 | 12 | 12 | 1 | 2 | 40 | 24 | 753 | | 328 |
| LSD.05 | 11 | 14 | 8 | 17 | 13 | 17 | 15 | 14 | 3 | 3 | 3 | 3 | 2 | 3 | 3 | 3 | 1481 | 452 | 1506 | | 656 |
| Trial H | 0.93 | 0.56 | 0.83 | 0.84 | 0.86 | 0.95 | 0.91 | 0.00 | 0.89 | 0.64 | 0.76 | 0.47 | 0.92 | 0.93 | 0.54 | 0.00 | 0.97 | 0.95 | 0.86 | | 0.72 |

DS dry season; WS wet season; NS non-stress; RS reproductive stage drought stress; DFF days to 50% flowering (days); PH plant height (cm); NT number of tillers; PL panicle length (cm); GYKGPH grain yield (kg ha^−1^); H heritability; IR74371-46-1-1 donor parent possesses *qDTY_12.1_;* IR96321-1447-561-B-1 possess *qDTY_1.1_* and *qDTY_3.1_*; Lalat recurrent parent, Sahabhagi dhan drought tolerant checks.
